# CFTR regulates B cell activation and lymphoid follicle development

**DOI:** 10.1186/s12931-019-1103-1

**Published:** 2019-07-01

**Authors:** Francesca Polverino, Bao Lu, Joselyn Rojas Quintero, Sara O. Vargas, Avignat S. Patel, Caroline A. Owen, Norma P. Gerard, Craig Gerard, Manuela Cernadas

**Affiliations:** 1 Asthma and Airway Disease Research Center, University of Arizona, Tucson, AZ 85718 USA; 2 Lovelace Respiratory Research Institute, Albuquerque, NM 87108 USA; 3 Division of Respiratory Diseases, Department of Medicine, Boston Children’s Hospital, Boston, MA 02115 USA; 4 Department of Pathology, Boston Children’s Hospital, Boston, MA 02115 USA; 5 Lahey Hospital and Medical Center, Burlington, MA 01805 USA; 6 Vertex Pharmaceuticals, Boston, MA 02210 USA; 7 Division of Pulmonary and Critical Care Medicine, Department of Medicine, Brigham and Women’s Hospital, Harvard Medical School, Boston, MA 02115 USA

**Keywords:** Cystic fibrosis, B lymphocyte, Lymphoid follicles, BAFF

## Abstract

**Background:**

Cystic fibrosis (CF) is an inherited disorder caused by mutations in the CF transmembrane conductance regulator (CFTR) gene that promotes persistent lung infection and inflammation and progressive loss of lung function. Patients with CF have increased lung lymphoid follicles (LFs) and B cell-activating factor of tumor necrosis factor family (BAFF) that regulates B cell survival and maturation. A direct role for CFTR in B cell activation and disease pathogenesis in CF remains unclear.

**Methods:**

The number of LFs, BAFF^+^, TLR4^+^ and proliferation marker Ki67^+^ B cells in lung explants or resections from subjects with CF and normal controls was quantified by immunostaining. The role of CFTR in B cell activation and LF development was then examined in two independent cohorts of uninfected CFTR-deficient mice (*Cftr* ^*−/−*^) and wild type controls. The number of lung LFs, B cells and BAFF^+^, CXCR4^+^, immunoglobulin G^+^ B cells was examined by immunostaining. Lung and splenocyte B cell activation marker and major histocompatibility complex class II (MHC class II) expression was quantified by flow cytometry. Inflammatory cytokine levels were measured in supernatants from isolated B cells from *Cftr* ^*−/−*^ and wild type mice stimulated in vitro with *Pseudomonas aeruginosa* lipopolysaccharide (LPS).

**Results:**

There was a significant increase in well-formed LFs in subjects with CF compared to normal controls. Increased B cell activation and proliferation was observed in lung LFs from CF subjects as was quantified by a significant increase in B cell BAFF, TLR4 and Ki67 expression. Uninfected *Cftr* ^*−/−*^ mice had increased lung LFs and BAFF^+^ and CXCR4^+^ B cells compared to wild type controls. Lung B cells isolated from uninfected *Cftr* ^*−/−*^ mice demonstrated increased MHC class II expression. In vitro, isolated B cells from *Cftr* ^*−/−*^ mice produced increased IL-6 when stimulated with LPS compared to wild type controls.

**Conclusions:**

These data support a direct role for CFTR in B cell activation, proliferation and inflammatory cytokine production that promotes lung LF follicle development in cystic fibrosis.

## Background

Cystic fibrosis (CF) is a monogenic autosomal recessive disorder associated with significant morbidity and mortality that affects approximately 1 in 3000 newborns in the US. Despite significant advances in treatment, the median predicted survival for a CF patient born between 2003 and 2007 is 37 years [1]. CF arises from mutations in the CF transmembrane conductance regulator (CFTR) that lead to impaired chloride and bicarbonate transport. Impaired CFTR epithelial cell function in patients with CF leads to viscous mucus, impaired mucociliary clearance and airway colonization with pathogenic bacteria, especially *Pseudomonas aeruginosa (P. aeruginosa)*. These changes in airway biology lead to progressive lung damage and respiratory insufficiency. Recently, the ionocyte was identified as the main CFTR expressing epithelial cell [2]. However, CFTR expression on both innate and adaptive immune cells has been increasingly appreciated to contribute to immune dysfunction and disease pathogenesis in CF.

The absence of normal CFTR expression on macrophages has been linked to increased inflammatory cytokine production, altered TLR4 trafficking and impaired resolution of infection and inflammation [3–5]. A direct role for CFTR in macrophage function was confirmed by siRNA knockdown of CFTR expression on human alveolar macrophages. Alveolar macrophages with silenced CFTR had increased IL-8 secretion, increased NF-κB phosphorylation and increased caveolin-1 expression [6]. The T helper 2 (Th2) skewing of CD4 lymphocytes from both patients and *Cftr* ^*−/−*^ mice has been well described [7, 8]. CFTR deficiency has also been linked to diminished regulatory CD4 T cell (Treg) effector function [9].

B cells are critical for adaptive immune responses and express CFTR mRNA. Human B cells that lack CFTR have impaired chloride conductance as is observed in CFTR-deficient epithelial cells [10, 11]. B cell-activating factor of tumor necrosis factor family (BAFF) is produced by B cells, T cells and myeloid lineage cells and plays an important role in B cell survival and maturation [12]. BAFF can bind to three receptors that are constitutively expressed on B cells (BAFF-receptor, transmembrane activator and calcium modulator and cyclophilin ligand interactor and B-cell maturation antigen). BAFF is not produced by B cells at steady state but is induced by antigen-activated helper T cells. BAFF produced by B cells can work in both an autocrine and paracrine manner [12–14]. The importance of BAFF in lung B cell development and immunity was recently reinforced and confirmed to be important in CF. Wild type and *Cftr* ^*−/−*^ mice treated with a neutralizing antibody that blocks BAFF resulted in B cell and lung CD4^+^ regulatory T cell (Treg) depletion. Blockade of BAFF and resultant B cell depletion increased the lung bacterial burden in both wild type and CFTR deficient mice infected with *P. aeruginosa*, although the bacterial load and lung resistance was higher in the CFTR deficient mice [15]. BAFF has been detected in bronchoalveolar lavage (BAL) fluid from patients with CF and was most elevated in patients infected with *P. aeruginosa*. BAFF was not detected in BAL fluid from healthy controls [16]. BAFF was also shown to be induced in the lungs of wild type mice infected with *P. aeruginosa* [16].

Peribronchial lymphoid follicles (LFs) have been observed in patients with CF and developed in wild type mice in response to bacterial infection. Wild type mice infected with *P. aeruginosa* had elevated levels of lung tissue BAFF and B cell chemoattractants including CXCL13 [16, 17]. Lung B cell BAFF expression has also been shown to correlate with LF development in chronic obstructive pulmonary disease (COPD) [13]. Lung BAFF and specifically autocrine B cell BAFF production may contribute to the promotion and persistence of airway inflammation as was demonstrated in patients with COPD [13]. These findings raise questions as to whether LF development may contribute to CF lung pathology. The observation of increased lung BAFF and LFs has been made in lung tissue from patients with CF that have airway colonization with pathogenic bacteria and BAL fluid and lung tissue from wild type mice infected with *P. aeruginosa* [16, 17]. However, a direct role for CFTR in B cell immune function has not been well characterized.

Several murine *Cftr* ^*−/−*^ lines deficient in CFTR have been developed and do not develop lung pathology in the absence of direct exposure to pathogenic bacteria [18]. However, age dependent increases in interstitial macrophages and interstitial thickening have been observed in lung tissue from uninfected *Cftr* ^*−/−*^ mice [19]. A different group examined uninfected *Cftr* ^*−/−*^ mice 16 to 20 weeks of age and also observed lung inflammatory cell infiltration that was not present in wild type controls. Interestingly, immunoglobulin chain genes were the genes that were most overexpressed in lung tissue from uninfected *Cftr* ^*−/−*^ mice versus wild type controls in this study [20]. These changes in unchallenged mice suggest that CFTR deficiency may contribute to lung inflammation in the absence of infection over time. Here, we demonstrate a role for CFTR in the promotion of tertiary lung LF development, B cell BAFF and CXCR4 expression and B cell inflammatory cytokine production in the absence of infection.

## Materials and methods

### Human lung sections

Lung tissue sections from CF patients were obtained from archival lung specimens from lung explants obtained at the time of lung transplantation or wedge resections/lobectomies performed for clinical indications. Normal lung tissue sections were identified by a clinical expert (S.O.V.) from surgical tissue obtained from patients without CF, bronchiectasis or other underlying primary lung disease. The control group was comprised of an uninvolved area of lung from a subjects with malignancy (peripheral nerve sheath tumor (*n* = 1), inflammatory myofibroblastic tumor (*n* = 1), pleomorphic sarcoma (*n* = 1), pulmonary carcinoid tumor (*n* = 1), anaplastic large cell lymphoma (*n* = 1)) and uninvolved lung tissue adjacent to an apical bleb (*n* = 5). The experimental protocol only used archival lung tissue and was approved by the institutional review board at Boston Children’s Hospital. Patient characteristics are described in Table 1. None of the subjects included in the study were on CFTR modulator therapy at the time of lung tissue sampling.

**Table 1:** Patient demographics

	Cystic fibrosis (*n* = 27)	Controls (*n* = 10)	*p* value
Age (yr)	15.9 ± 0.98	15.3 ± 0.65	0.71
Sex (M/F)	15/12	5/5	0.77
Race or ethnic group (no.)			
Caucasian	25		
Hispanic	1		
Native American	1		
Body-mass index (kg/m^2^)	18.1 ± 2.2		
Prebronchodilator FEV_1_ (% of predicted value)	33.0 ± 0.04		
Pancreatic insufficiency (no.)	27/27		
Airway colonization with:			
*Pseudomonas aeruginosa*	19/25		
Methicillin-resistant *Staphylococcus aureus*	8/25		
Methicillin-sensitive *Staphylocccus aureus*	9/25		
*Burkholderia cepacia* complex	6/25		
*Stenotrophomonas maltophilia*	7/25		
*Escherichia coli*	3/25		
*Enterobacter* species	1/25		
*Klebsiella* species	1/25		
Other *Staphylococcus* species	1/25		
CFTR genotype			
Homozygous F508del-CFTR	14		
Heterozygous F508del-CFTR	4		
Heterozygous other	1		
Unknown	8		
Pancreatic insufficiency (no.)	27/27		
CF-related diabetes mellitus (no.)	13/27		
CF-related liver disease (no.)	5/27		
Non-tuberculous mycobacteria lung infection (no.)	1/21		
Allergic bronchopulmonary aspergillosis (no.)	4/27		
Immunoglobulin E (mg/dL)	278 ± 107.5		

Plus-minus values indicate ± SEM

*CF* cystic fibrosis, *CFTR* cystic fibrosis transmembrane conductance regulator, *F508del-CFTR* delta 508 CFTR mutation, *FEV*_*1*_ forced expiratory volume in 1 s

### Murine Cftr ^−/−^ models

All studies were conducted in accordance with the Institutional Animal Care and Use Committees of Boston Children’s Hospital and Brigham and Women’s Hospital/Harvard Medical School. The generation, characterization and maintenance of the Cftr^tm1UNC^ mouse (*Cftr* ^*−/−*^) congenic on C57BL/6 J background has been previously described [21]. The first cohort included *Cftr* ^*−/−*^ and C57BL/6 J control mice 18–20 weeks of age housed at Brigham and Women’s Hospital (cohort 1). Lung tissue that was fixed with formalin and embedded in paraffin was available from this cohort. The second cohort included Cftr^tm1UNC^ and C57BL/6 J control mice between 43 and 59 weeks of age, which were generously provided by Dr. Mitchell Drumm and Case Western Reserve University CF Mouse Models Core (cohort 2). These mice were euthanized with overdose of pentobarbital, and lung and splenic tissue harvested within 48 h of arrival at the Boston Children’s Hospital animal facility. Briefly, after perfusion of the right ventricle with PBS, one lung was isolated and removed for isolation of mononuclear cells. The contralateral lung was then inflated with and fixed in formalin for immunohistochemistry as previously described [13].

### Characterization of pulmonary LFs using immunofluorescence

Human cohort: Formalin-fixed and paraffin-embedded (5 μm-thick) lung sections from explants or lung resections from patients with CF (*n* = 27) or normal lung tissue obtained during surgical resections (*n* = 10) were deparaffinized, and antigen retrieval was performed by boiling the slides immersed in 0.01 M sodium citrate and 2 mM citrate buffer (pH 6.0) in a microwave. To identify human pulmonary LFs, lung sections were immunostained with: 1) murine anti-CD20 IgG conjugated to Alexa-Cy5 IgG; 2) rat anti-BAFF IgG conjugated with Alexa 488 F (ab’)_2_; and 3) rabbit anti-ki67 conjugated with Alexa 546 IgG. All the primary antibodies were purchased from Abcam (Cambridge, MA) and the secondary antibodies were purchased from Invitrogen.

Murine cohorts: Formalin-fixed and paraffin-embedded sections (5 μm thick) of lung sections were obtained from two cohorts of wild type and *Cftr* ^*−/−*^ mice housed at different institutions and animal facilities (see above). Lung sections were incubated with rabbit anti-CD45R or murine anti-CD20 to identify B cells and were additionally stained with rat anti-BAFF, rabbit anti-IgG and goat anti-CXCR4 primary antibodies purchased from Abcam. Goat anti-rat Alexa 488 F (ab’)_2_, goat anti-mouse Alexa Cy5 IgG and goat anti-rabbit Alexa 546 IgG secondary antibodies were obtained from Invitrogen. For TLR4 analysis, rabbit anti-CD20 antibody (Abcam), mouse anti-TLR4 antibody (Abcam) and goat anti-mouse IgG AlexaFluor-488 and goat anti-rabbit AlexaFluor-546 secondary antibodies (Invitrogen) were used. Slides were quenched with Sudan black and counterstained with 4′,6-diamidino-2-phenylindole (DAPI) as previously described [13]. Lung sections were also immuno-stained with the appropriate isotype- and species-matched non-immune control antibodies.

### Microscopy and image analysis

For each slide, a minimum of 20 randomly-selected high-magnification fields were evaluated using a Leica epi-fluorescence microscope (Leica Microsystems, Buffalo Grove, IL) except in the case of TLR4 analysis where a Nikon Eclipse 80i microscope (Melville, NY) was used. In both human and murine cohorts, the number of LFs, CD20- or CD45R-positive B cells, BAFF-, CXCR4-, IgG-, TLR4- or Ki67-positive B cells were counted, and the data were expressed as median number/tissue area. Lymphoid follicles were defined as aggregates containing more than 40 mononuclear cells exhibiting the typical topographical arrangement with centrally located B cells along with a few follicular dendritic cells and CD4 and CD8 cells in the periphery. LFs located within a clearly defined lymph node capsule were excluded from analysis. Images of the immunostained lung sections were captured and analyzed using a confocal microscope (Olympus Corporation, Center Valley, PA). Confocal micrographs were recorded under fluorescence imaging mode in which cells were exposed to 488, 570, and 670 nm light attenuated by an acusto-tunable optical filter. Tissue area was measured using MetaMorph software (Molecular Devices, Sunnyvale, CA). The area of the LFs was also measured using MetaMorph software by drawing a region of interest around the peripheries of the LFs between the B-cell-abundant areas and areas in which there were few B-cells [13].

### Lung and splenocyte mononuclear cell flow cytometry

Flow cytometry was performed on mononuclear cells isolated from spleen and lung tissue from wild type and *Cftr* ^*−/−*^ mice. Lungs and spleen were harvested and digested as previously described [22]. Briefly, minced spleen tissue was passed over nylon mesh filter and minced lung tissue was digested with collagenase (Type IV, Worthington, Lakewood, NJ) and DNAse I (Roche/Sigma) for 1 h at 37 °C with constant horizontal shaking to generate single cell suspension as previously described [22]. After red cell lysis, the single cell suspensions were incubated with purified rat anti-mouse CD16/CD32 (BD Biosciences, San Diego, CA) to block Fcγ receptors for 30 min at 4 °C in flow cytometry buffer (PBS with 2.5% fetal bovine serum albumin (Gibco/Thermo Fisher Scientific, Waltham, MA). Cells were subsequently stained with optimal concentrations of conjugated monoclonal antibodiess and their appropriate isotype controls for 30 min at 4 °C. Directly conjugated fluorochrome antibodies were used including anti-mouse PE-Cy7 CD19 antibody (Biolegend, San Diego, CA), anti-mouse FITC MHC class II antibody (I-A/I-E, BD Biosciences), anti-mouse PE CD86 antibody (BD Biosciences), anti-mouse PE CD8α antibody (BD Biosciences), anti-mouse PE CD4 antibody (BD Biosciences), anti-mouse PE-Cy7 CD45 antibody (Thermo Fisher Scientific), anti-mouse FITC CD3 antibody (BD Biosciences) and propidium iodide (Sigma Aldrich, St. Louis, MO). The cells were then washed and analyzed using a FACS Canto II flow cytometer (Becton Dickinson, San Jose, CA) and Flow Jo software (Ashland, OR). After doublet exclusion, propidium iodide staining was used to exclude dead cells. Results are expressed as the median fluorescent intensity (MFI) index. MFI index is the MFI with the tested antibody divided by the MFI of the appropriate isotype-matched control antibody.

### B cell functional assays

Untouched B cells were isolated from mononuclear splenocyte cell suspensions from *Cftr* ^*−/−*^ and wild type mice with mouse B cell Isolation Kit (Miltenyi Biotec, Auburn, CA) as per manufacturer’s instructions. 1 × 10^5^ B cells in Dulbecco’s Modified Eagle’s Medium (DMEM; Life Technologies, Grand Island, NY) containing 10% fetal bovine serum (Gibco/Thermo Fisher Scientific), 100 IU/ml penicillin and 100 mg/ml penicillin/streptomycin (Gibco/Thermo Fisher Scientific) were plated in 96-well plates and stimulated with media or 25 μg/ml lipopolysaccharide (LPS, from *Pseudomonas aeruginosa*, Sigma Aldrich) at 37 °C and 5% CO_2_ [23] Supernatants were harvested at 48 h and stored at -80 °C until further analysis. Murine IL-6 and IL-10 were measured in supernatants by ELISA as per manufacturer’s instructions (ThermoFisher Scientific).

### Statistics

For pairwise comparisons, parametric and nonparametric data were analyzed using two-sided Student’s t tests and Mann-Whitney U tests, respectively. Bar graphs represent mean and SEM, whereas boxes in box plots show the median values and 25th and 75th percentiles, and error bars show the 10th and 90th percentiles. Correlation coefficients were calculated using the Pearson rank method. *P* value less than or equal to 0.05 was considered statistically significant. Analyses were performed using GraphPad prism (GraphPad Software, San Diego, CA).

## Results

### Patient characteristics

Lung tissue sections were obtained from patients with CF (*n* = 27) and normal controls (*n* = 10). The majority of the CF lung tissue came from lung explants at the time of transplantation. Age and gender matched controls were also analyzed. Patient characteristics are listed in Table 1. The majority of the patients had severe lung disease as defined by forced expiratory volume in 1 s (FEV1) percent predicted. The average FEV1 was 33.0% predicted. 76.0% percent had airway colonization with *P. aeruginosa* and 73.6% were homozygous for the F508del-CFTR mutation. Reflective of the severity of the patients in this cohort, BMI was reduced and all the patients were pancreatic insufficient. 48.1% of patients had CF-related diabetes mellitus. The average age was 15.9 and males and females were equally represented. The normal controls were of similar age and gender. Pulmonary function testing data were not available for normal controls.

### Pulmonary LFs were increased in patients with CF

**Fig. 1:**
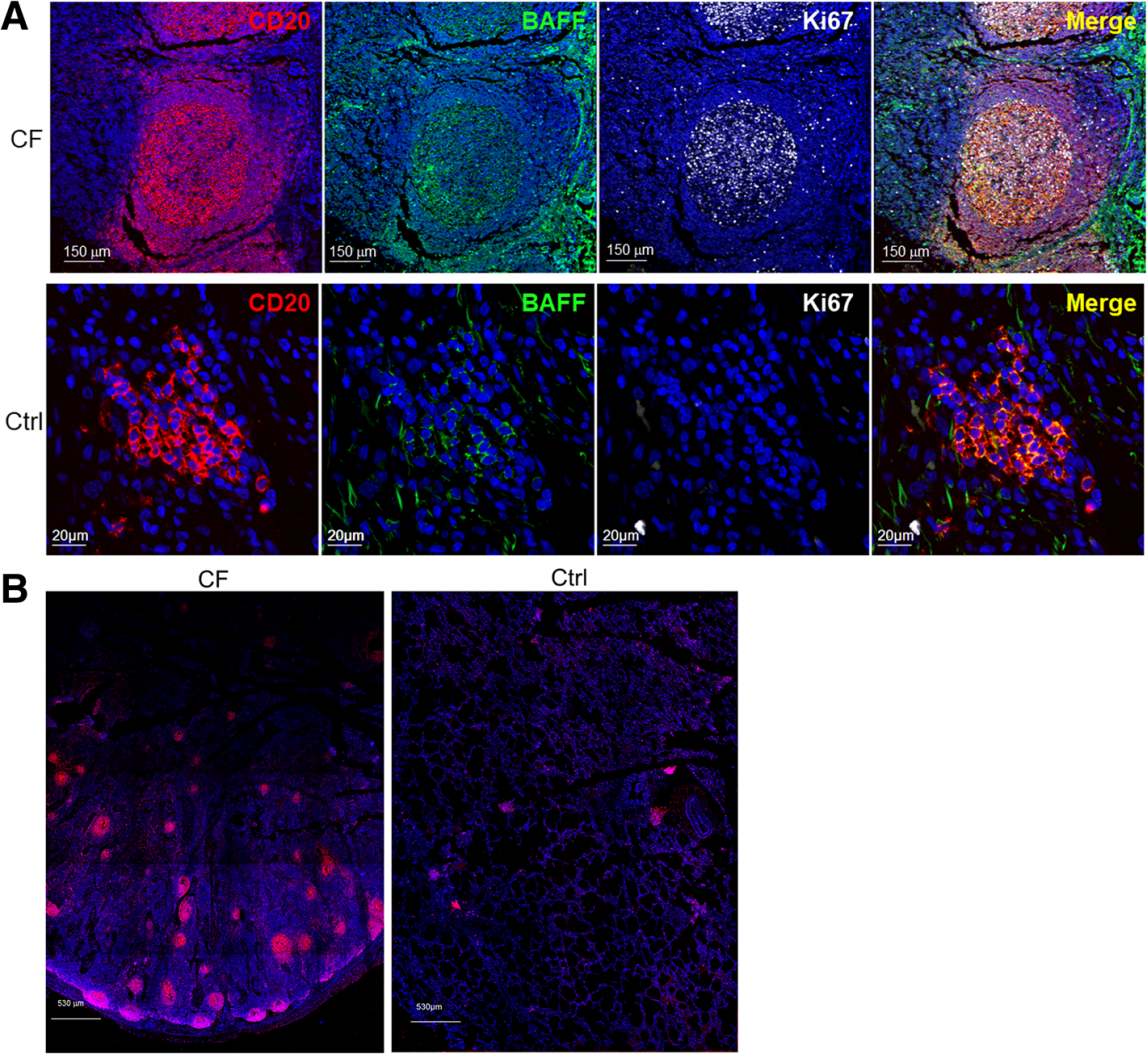
Lung lymphoid follicles, B cells, BAFF^+^ and Ki67^+^ B cells were increased in CF patients. **a** Representative confocal images of triple-color immunofluorescence staining of representative pulmonary lymphoid follicles (LFs) from subjects with CF (top panels) and normal controls (Ctrl, bottom panels). B cells are identified by staining with red fluorophore for CD20. BAFF is identified by staining with green fluorophore. Ki67 positive cells have a gray color. 4′6-diamidino-2-phenylindole (blue) was used to counterstain the nuclei. The final panel in each row is a merged file of CD20, BAFF and Ki67 staining. In the CF images (top row), the magnification is 20X. In the control images (bottom row), the magnification is 60X. However, for the control images digital zooming was used to better visualize the CD20^+^ cells. The images shown are representative of LFs in CF subjects (*n* = 27) and normal controls (*n* = 10). **b** Representative low power images of lung tissue from a subject with CF (left panel) and normal control (right panel). Lymphoid follicles are identified by staining with red fluorophore for CD20. 4′6-diamidino-2-phenylindole (blue) was used to counterstain the nuclei

**Fig. 2:**
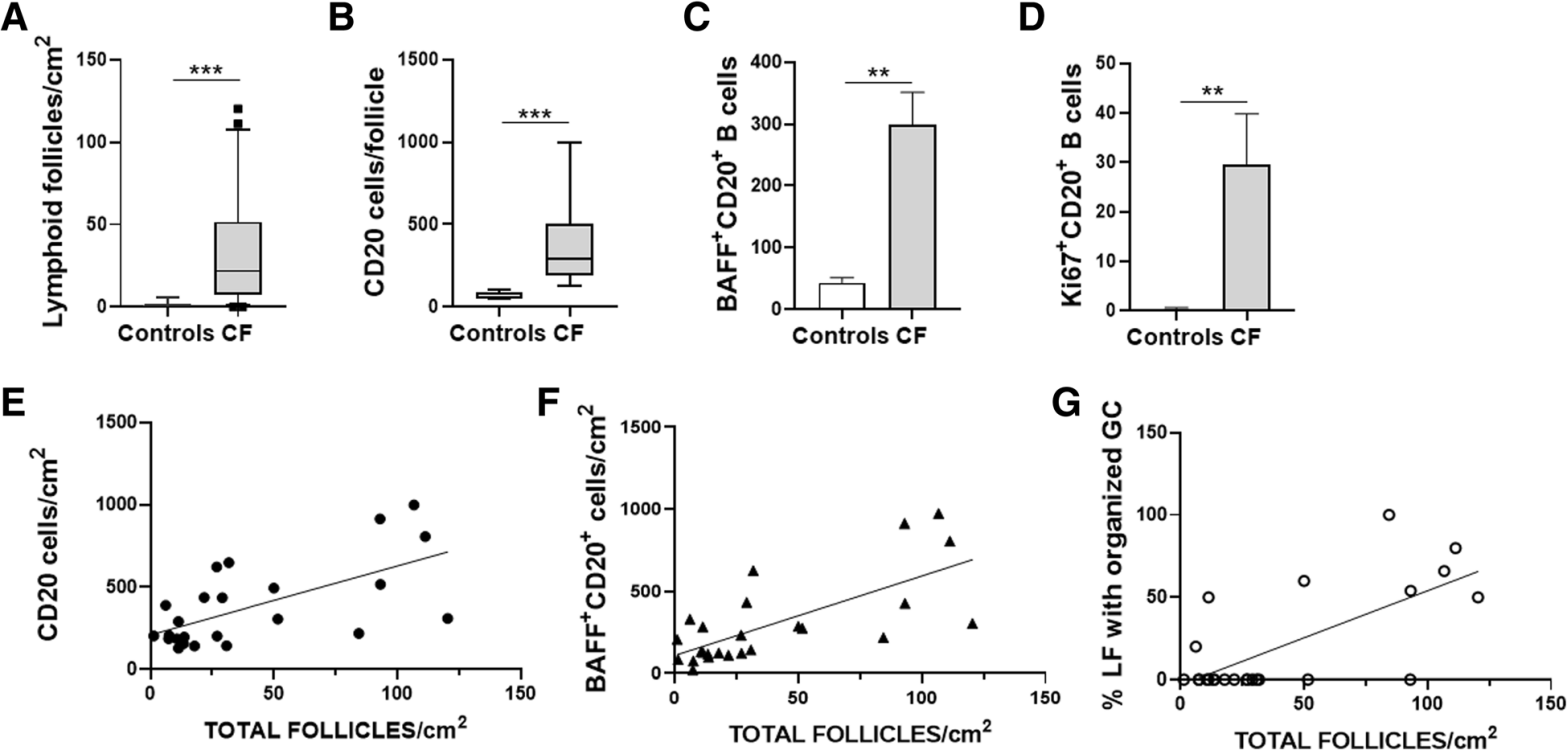
B cells and BAFF^+^ and Ki67^+^ B cells were increased in patients with CF**.** The number of **a** lymphoid follicles, **b** B cells, **c** BAFF^+^ and **d** Ki67^+^ B cells were quantified in lung tissue from CF subjects (*n* = 27) and normal controls (*n* = 10). There was significant increase in LFs, B cells identified by CD20 staining, BAFF^+^ and Ki67^+^ B cells in subjects with CF compared to controls. B cells, BAFF^+^ B cells and lymphoid follicles (LFs) with organized germinal centers strongly correlated with total LFs. There was a significant correlation between the number of lymphoid follicles and **e** CD20-positive B cells (*R*^2^ = 0.411, *p* = 0.006), **f** BAFF-positive B cells (*R*^2^ = 0.498, *p* < 0.0001) and **g** LFs with organized germinal centers (*R*^2^ = 0.497, *p* < 0.0001) in patients with CF. This is a quantitative assessment of the data demonstrated in the representative images in Fig. 1. Mann-Whitney U test was used to perform the statistical analysis (**a**-**d**). Box plots show the median values and 25th and 75th percentiles, and error bars show the 10th and 90th percentiles. Squares in (**a**) indicate outliers. Correlation coefficients were calculated using the Pearson rank method. A total of 27 CF subjects were analyzed. *p ≤ 0.05; **p ≤ 0.001; ***p ≤ 0.0001, CF versus normal controls

LFs were quantified in lung tissue sections obtained from patients with CF and normal controls. There was a significant increase in the number of LFs in lung tissue from patients with CF compared with controls (37.9 ± 7.4 total LFs/cm^2^ to 1.45 ± 0.7 total LFs/cm^2^) (Figs. 1a, b and 2a). Consistent with the composition of LF, the number of CD20^+^ cells was also significantly higher in lung LF from patients with CF compared to controls (373.6 ± 50.4 CD20^+^ B cells to 66.8 ± 10.7 CD20^+^ B cells) (Figs. 1a, b and 2b). The number of B cells correlated with the significantly larger and well-organized LFs in CF subjects compared to controls (Fig. 2e). Of note, the images obtained from CF subjects were taken at a higher magnification as the LFs were too large to capture on lower magnification (Fig. 1a). Please note size markers on images. The control lung LFs were significantly smaller and less organized (Fig. 1a and b). Figure 1b is a composite image of an area of lung tissue at low magnification that highlights the large number and size of LFs in patients with CF. The clusters of CD20 staining in red identify individual LFs. No statistically significant differences in follicle or B cell numbers were observed when *Pseudomonas aeruginosa* (PSA)-colonized subjects were compared to non-PSA colonized subjects or between patients with and without metabolic dysfunction.

### LF B-cell BAFF and Ki67 expression is increased in human CF lungs

Given the importance of BAFF in promoting B cell survival, maturation and augmenting the adaptive immune response, BAFF expression in lung LFs in CF and controls was also examined. BAFF expression was significantly higher on LF B cells from patients with CF compared to controls (298.9 ± 52.9 BAFF^+^ B cells to 43 ± 8.3 BAFF^+^ B cells) (Fig. 2c). The number of BAFF^+^ B cells correlated with the number of LF (Fig. 2f) consistent with increased B cell activation in CF. Ki67 is a reliable proliferation marker and was used to identify B cells in a proliferative state. There were a greater number of Ki67^+^ B cells in CF LF compared to controls (29.66 ± 10.2 Ki67^+^ B cells to 0.3 ± 0.3 Ki67^+^ B cells)(Fig. 2d). There was a statistically significant correlation between BAFF and Ki67 expression (data not shown). Together these findings suggest lung LF B cells in CF have increased proliferative capacity and ability to augment B cell responses.

### LF with organized germinal centers correlated with LF numbers in human CF lungs

One striking feature of many of the LF in patients with CF was the presence of well-developed germinal centers with centroblasts. No similar areas were observed in the normal controls. The percentage of LF with organized germinal centers correlated with LF numbers (Fig. 2g).

### Lung LFs are increased in uninfected Cftr^−/−^ lungs

**Fig. 3:**
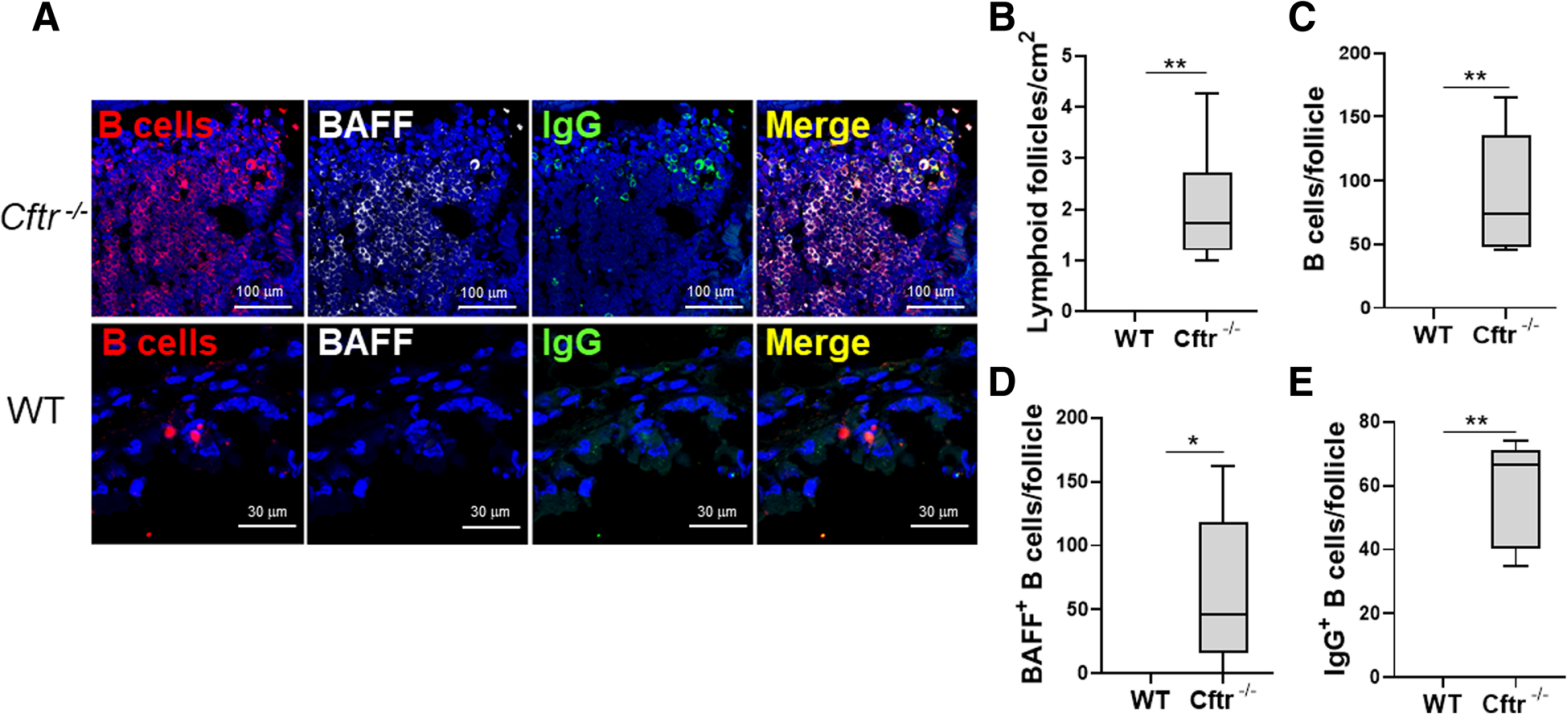
LFs, B cells, BAFF^+^ and IgG^+^ B cells were increased in uninfected Cftr ^−/−^ mice. **a** Representative confocal images of triple-color immunofluorescence staining of representative pulmonary LFs from uninfected Cftr ^−/−^ mice (top two rows) and wild type (WT) controls (bottom row) from cohort 1. B cells are identified by staining for CD45R and red fluorophore. BAFF positive cells have a grey color. IgG positive B cells were identified by staining with green fluorophore. 4′6-diamidino-2-phenylindole (blue) was used to counterstain the nuclei. The final panel in each row is a merged file of CD45R, BAFF and IgG staining. In the Cftr ^−/−^ mice images (top row), the magnification is 60X. In the wild type control images (bottom row), the magnification is 100X. The images shown are representative of LFs in Cftr ^−/−^ mice (*n* = 6) and WT controls (*n* = 7). Cftr ^−/−^ and WT lung **b** Lymphoid follicles, **c** B cells, **d** BAFF^+^ B cells and **e** IgG^+^ B cells were quantified. Mann-Whitney U test was used to perform the statistical analysis (**b**-**e**). Box plots show the median values and 25th and 75th percentiles, and error bars show the 10th and 90th percentiles. *p ≤ 0.05, **p ≤ 0.001, Cftr ^−/−^ mice versus WT

**Fig. 4:**
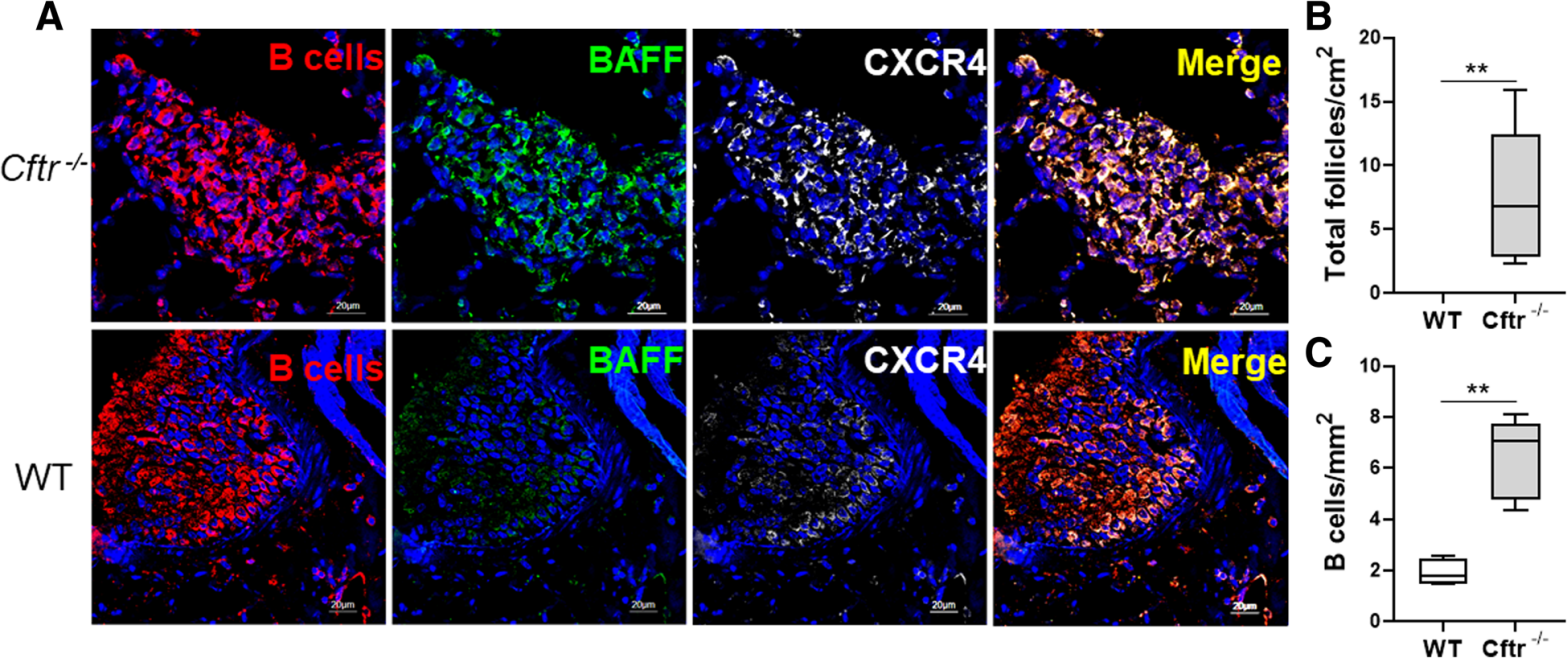
LFs, B cells, BAFF^+^ and CXCR4^+^ B cells were increased in uninfected Cftr ^−/−^ mice**.** A second cohort of uninfected Cftr ^−/−^ mice bred, housed and maintained at a different facility were also analyzed (cohort 2). **a** Representative confocal images of triple-color immunofluorescence staining of representative pulmonary LFs from Cftr ^−/−^ mice (top row) and wild type (WT) controls (bottom row). B cells are identified by staining for CD20 and red fluorophore. BAFF positive B cells were identified by staining with green fluorophore. CXCR4 positive B cells have a grey color. 4′6-diamidino-2-phenylindole (blue) was used to counterstain the nuclei. The final panel in each row is a merged file of CD20, BAFF and CXCR4 staining. In both the WT and Cftr ^−/−^ images the magnification is 60X. The images shown are representative of LFs in Cftr ^−/−^ mice (*n* = 8) and WT controls (*n* = 4). Cftr ^−/−^ and WT lung **b** Lymphoid follicles and **c** B cells were quantified. Mann-Whitney U test was used to perform the statistical analysis (**b**-**c**). Box plots show the median values and 25th and 75th percentiles, and error bars show the 10th and 90th percentiles. **p ≤ 0.005, Cftr ^−/−^ mice versus WT

Pulmonary LFs were quantified in uninfected *Cftr*^*−/−*^ mice and wild type controls. There was a statistically significant increase in the number of lung LFs in *Cftr*^*−/−*^ compared to wild type control (2.04 ± 0.4 LFs/cm^2^ to 0 ± 0.0 LFs/cm^2^) in the first *Cftr*^*−/−*^ cohort housed at Brigham and Women’s Hospital (Fig. 3a and b). Given the potential role for microenvironmental, housing and microbiome influences to alter lung immune development, we examined a second cohort of uninfected *Cftr*^*−/−*^ mice bred and housed at a different facility, Case Western University. We observed a similar statistically significant increase in lung LF in uninfected *Cftr*^*−/−*^ mice compared to wild type (7.67 ± 1.8 LFs/cm^2^ to 0.57 ± 0.57 LFs/cm^2^)(Fig. 4a and b).

### LF B-cell BAFF and CXCR4 expression is increased in uninfected Cftr^−/−^ lungs

Despite the absence of an exogenous infectious stimulus, lung LF B cells from *Cftr*^*−/−*^ mice had significantly increased BAFF expression. There was essentially no BAFF expression on wild type lung LF B cells (Fig. 3a and d). These findings were validated in the second uninfected *Cftr*^*−/−*^ murine cohort (Fig. 4a). CXCR4 is a critical chemokine receptor in B cell development. CXCR4 B cell expression is dynamically regulated during B cell maturation [24, 25]. CXCR4 is also an important centroblast marker in both humans and mice [25]. There was a significant increase in CXCR4 staining throughout the lung LFs in *Cftr*^*−/−*^ mice compared to wild type (Fig. 4a). In wild type mice, CXCR4 staining was clustered to an area of the LF and was less intense (Fig. 4a). Immunoglobulin G (IgG) staining was also performed and was significantly increased in uninfected *Cftr*^*−/−*^ LF B cells (Fig. 3e). The intensity of IgG staining correlated with intensity of BAFF staining as was demonstrated by triple staining (Fig. 3a, merge panel).

### MHC class II expression is increased in lung B cells from Cftr^−/−^ mice

**Fig. 5:**
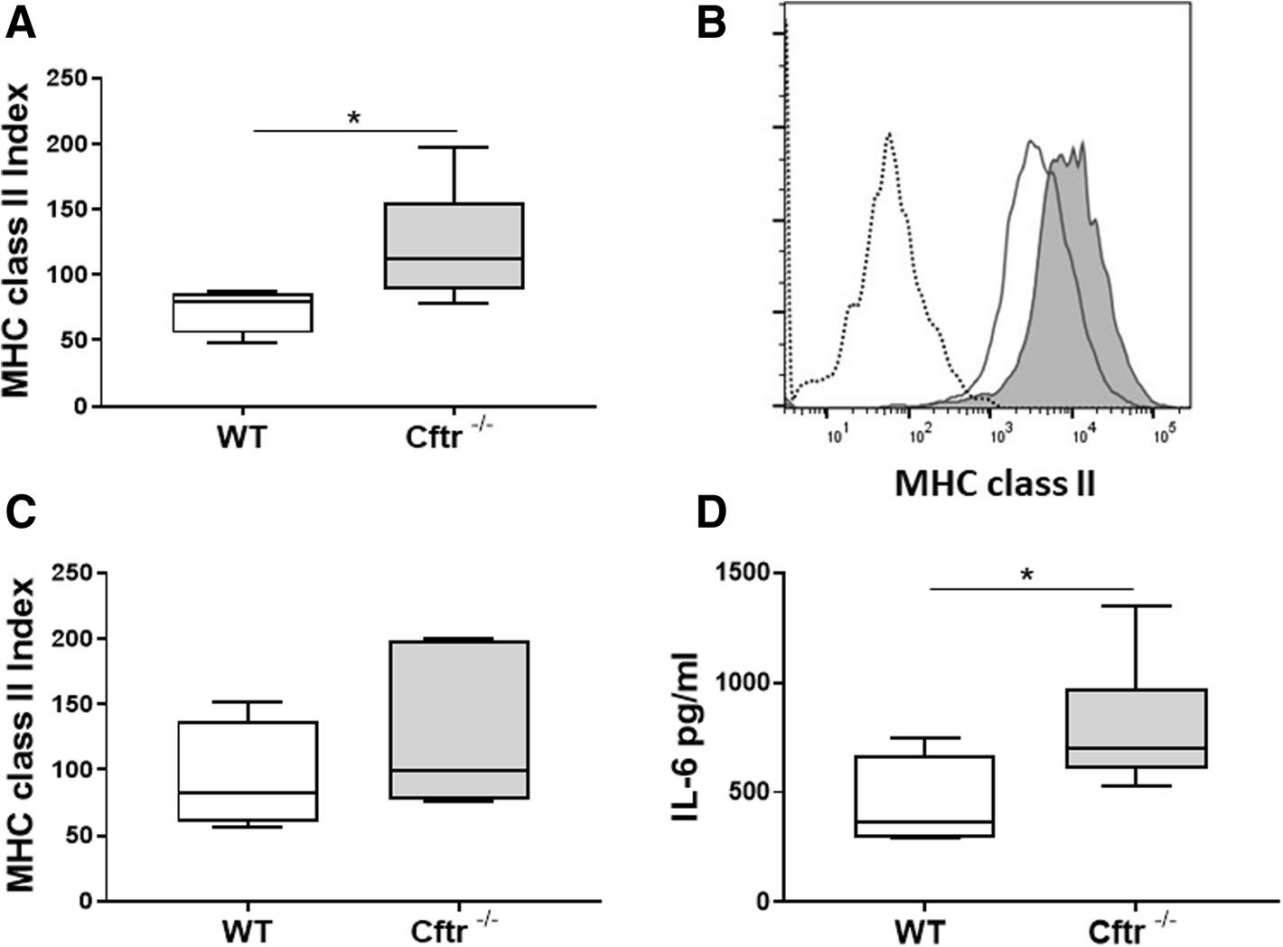
Cftr ^−/−^ B cells have increased MHC class II and increased IL-6 response to LPS MHC class II expression was measured on B cells from lung mononuclear cells **a** isolated from uninfected Cftr^−/−^ and wild type controls with directly conjugated antibodies by flow cytometry. **b** A representative histogram of MHC class II expression on lung B cells from uninfected Cftr ^−/−^ mice (gray histogram) and wild type controls (white histogram). Isotype control staining is represented by dotted line. MHC class II expression was also measured on B cell splenocytes isolated from uninfected Cftr ^−/−^ mice and wild type controls (**c**). **d** IL-6 production from B cells isolated from the spleens of Cftr ^−/−^ (*n* = 7) and WT mice (*n* = 4) stimulated with media or lipopolysaccharide (LPS) for 48 h was determined. The concentration of IL-6 in supernatants was determined by ELISA. Box plots show the median values and 25th and 75th percentiles, and error bars show the 10th and 90th percentiles. *p ≤ 0.05, Cftr ^−/−^ mice versus WT.

Lung and splenic B cells were also analyzed by flow cytometry. One lung from each mouse was disaggregated and digested to release mononuclear cells that were stained with directly conjugated antibodies. No differences in the percentage of B, CD4 or CD8 T cell cells were observed between *Cftr*^*−/−*^ lung and spleen compared to wild type controls (data not shown). This is consistent with prior publication of B and T lymphocyte subsets in *Cftr*^*−/−*^ mice on C57BL/6 background [26]. B cell activation was assessed by staining with CD86 and major histocompatibility complex class II (MHC class II). There were no differences in CD86 costimulatory cell marker expression between *Cftr*^*−/−*^ and wild type B cells from either lung or spleen (data not shown). However, there was a statistically significant increase in MHC class II expression on *Cftr*^*−/−*^ lung B cells compared to controls (Fig. 5a and b). There was no significant difference in MHC class II expression on splenic B cells (Fig. 5c).

### B cells isolated from Cftr^−/−^ mice have increased IL-6 production in response to LPS

Murine splenocyte B cells were stimulated with media or LPS for 48 h. Supernatants were harvested and IL-6 and IL-10 levels determined by ELISA. Inflammatory cytokines were produced upon stimulation with LPS. *Cftr*^*−/−*^ B cells produced significantly more IL-6 in response to LPS stimulation then wild type controls (Fig. 5d). Neither wild type nor Cftr^*−/−*^ mice produced IL-6 in the absence of LPS stimulation. There was no significant IL-10 production from either *Cftr*^*−/−*^ or wild type control B cells in response to the above conditions (wild type: 298.8 pg/ml-media control, 274.2 pg/ml-LPS; Cftr^−/−^: 284.4 pg/ml-media control, 289.5 pg/ml-LPS).

### LF B-cell TLR4 expression is increased in human CF lungs

**Fig. 6:**
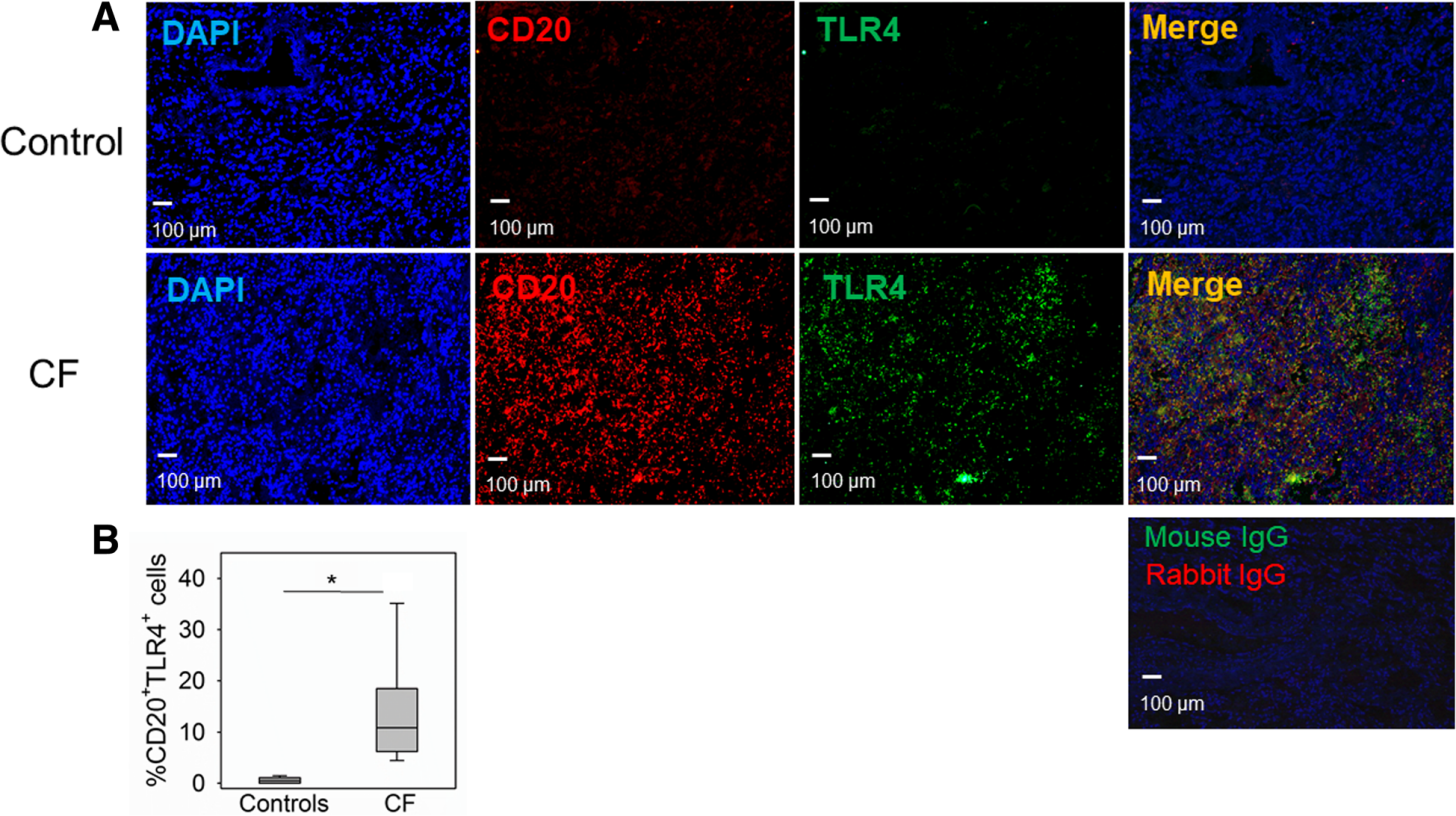
TLR4^+^ B cells are increased in patients with CF. **a** Representative confocal images of triple-color immunofluorescence staining of representative pulmonary lymphoid follicles (LFs) from subjects with CF (bottom panels) and normal controls (top panels). B cells are identified by staining with red fluorophore for CD20. TLR4 is identified by staining with green fluorophore. 4′6-diamidino-2-phenylindole (blue) was used to counterstain the nuclei. The final panel in each row is a merged file of CD20, TLR4 and DAPI staining. The percentage of TLR4^+^ B cells was quantified in lung tissue from CF subjects (*n* = 7) and normal controls (*n* = 5). There was significant increase in TLR4^+^ B cells in subjects with CF compared to controls (**b**). Images were taken at 10X using a Nikon Eclipse 80i microscope. Mann-Whitney U test was used to perform the statistical analysis. Box plots show the median values and 25th and 75th percentiles, and error bars show the 10th and 90th percentiles. *p = 0.003, CF versus normal controls

One potential mechanism by which CFTR deficiency could alter B cell function is through interactions with other molecules such as TLR4 that can interact with many cellular pathways and has been shown to interact synergistically with BAFF [27]. CFTR has also been linked to TLR4 signaling in macrophages [28]. TLR4 is not constitutively expressed on human B cells. However, it has been shown to be upregulated in inflammatory states [29]. TLR4 expression in lung LFs in CF and controls was examined. TLR4 expression was significantly higher on LF B cells from patients with CF compared to controls (10.8% ± 10.5 TLR4^+^ B cells to 0.49% ± 0.61 TLR4^+^ B cells) (Fig. 6). These findings suggest lung LF B cells in CF express TLR4 that may amplify responses to bacterial pathogens and promote B cell dysregulation.

## Discussion

CFTR deficiency on epithelial cells drives alterations in airway biology that promote infection and ineffective airway clearance that lead to the progressive loss of lung function in patients with CF. Patients with identical clinical characteristics and CFTR mutations can have very disparate outcomes. Individual differences in immune responses and maintenance of homeostasis may play a critical role in disease progression and morbidity, independent of CFTR deficiency. However, the findings presented here and prior work in other cell types points to a direct role for CFTR deficiency on immune function in CF that may alter clinical outcomes.

Tertiary LFs can develop in the setting of chronic inflammation and/or infection [30, 31]. Lung tissue from patients with CF had numerous tertiary LFs, significantly more than the controls. The majority of the LFs were well organized with well-defined germinal centers (Fig. 1a and b). In contrast to secondary lymphoid organs, tertiary LFs are not encapsulated and are directly exposed to local stimuli, antigens and inflammatory cytokines [30]. The proximity of these LFs to infectious pathogens may facilitate protective immune responses. However, in the case of CF where patients are chronically infected with bacteria that are not eradicated by antibiotics, their proximity may also lead to a feedback loop of inflammatory mediators, leukocyte recruitment and immune activation, which in turn could promote aberrant immune responses.

It is challenging in the setting of chronic infection to tease out what aspects of altered lung immunity are a direct effect of CFTR deficiency and which are driven by chronic infection and immune activation. Prior work has observed lung LF development in wild type mice infected with *P. aeruginosa* [17]. In order to determine the role of CFTR in the development of tertiary LFs and B cell responses, lung tissue from uninfected *Cftr* ^*−/−*^ and wild type mice was examined here. Lung tissue from uninfected *Cftr* ^*−/−*^ mice had a significantly higher number of LFs compared to wild type controls (Fig. 3a and b). To validate these findings, a second cohort of mice was obtained from a collaborator at a different institution exposed to a different microenvironment and microbiome. We observed a similar increase in the number of pulmonary LFs in uninfected *Cftr* ^*−/−*^ mice compared to controls (Fig. 4a and b). Despite the fact that the *Cftr* ^*−/−*^ mouse does not recapitulate the phenotype of lung disease observed in humans, there have been reports in the literature of changes in the lungs of uninfected *Cftr* ^*−/−*^ mice including lung inflammatory cell infiltration [19, 20]. Interestingly, the observations were made in mice that were 16 to 24 weeks of age and in the case of Kent, G. et al., were age dependent. It is possible that organized LFs make take time to develop in the absence of infection in *Cftr* ^*−/−*^ mice.

The potential important role for age in the development of lymphoid follicle development is also highlighted by the higher number of follicles in the older cohort (cohort 2) compared to cohort 1 (Figs. 3b and 4b). However, differences in environmental or microbiome exposures between the cohorts cannot be excluded. Intestinal isolated lymphoid follicle development has also been shown to significantly increase with age [32]. As patients with cystic fibrosis achieve longer lifespans, the cumulative effect of immune dysfunction superimposed on the age-related immune senescence may play a role in disease progression.

One of the likely drivers of LF development in the lung is BAFF as has been previously shown in COPD [13]. We observed a marked increase in BAFF expression in uninfected *Cftr* ^*−/−*^ lung LF B cells (Figs. 3a, d and 4a), which may contribute to B cell survival and may drive other autocrine and paracrine effects [12]. BAFF and tertiary lung lymphoid structures have been shown to have detrimental effects. This was demonstrated in a murine model of COPD where BAFF was blocked by soluble BAFF fusion protein. In addition to reduced inflammation and the prevention of LF development, there was a significant reduction in alveolar wall destruction [33]. Depletion of BAFF in *Cftr* ^*−/−*^ mice prior to infection with *P. aeruginosa* depleted B cells and impaired antimicrobial immunity. The mice were euthanized 3 days after infection. LF development was not examined in this study that highlights the importance of effective humoral immunity in CF [15]. However, aberrant or excessive BAFF expression could amplify inflammation with deleterious effects. The fact that B cell BAFF and Ki67 expression was increased in lung LFs that were also increased in number in uninfected *Cftr* ^*−/−*^ mice suggests a direct role for CFTR deficiency in increased BAFF expression, B cell activation and the promotion of LF development.

To further characterize the effects of CFTR deficiency on B cell phenotype and function, lung and splenic B cells from *Cftr* ^*−/−*^ mice were analyzed by flow cytometry and stimulated in vitro. There were no differences in costimulatory molecule expression or the percentage of B cells. However, there was a statistically significant increase in MHC class II expression on *Cftr* ^*−/−*^ lung B cells compared to wild type (Fig. 5a and b). MHC class II expression on B cells is critical for antigen presentation. In the presence of large amounts of antigen, B cells in tertiary LFs could present antigen to other lymphocytes, which may be further amplified by increased MHC class II expression [30]. It will remain to be determined if the increase in MHC class II expression is an indirect consequence of an increase in the activation state of B cells in *Cftr* ^*−/−*^ mice or if CFTR deficiency plays a direct role in regulating MHC class II expression. In vitro, *Cftr* ^*−/−*^ splenic B cells produced more IL-6 when stimulated with LPS than wild type controls (Fig. 5d). IL-6 has been shown to promote lung LF development [34]. Augmented IL-6 inflammatory responses to chronic airway infection may be an important mechanism that amplifies LF formation in CF and may work synergistically with BAFF that is increased in lung LF B cells from uninfected *Cftr* ^*−/−*^ mice. Increased IL-6 mRNA was observed in bronchoalveolar lavage cells stimulated with LPS from mice deficient in myeloid lineage CFTR [5], which suggests altered cytokine responses and IL-6 production may be present in other cell lineages in the absence of CFTR. These data suggest an important role for CFTR promoting a pro-inflammatory B cell phenotype, which in turn could promote tertiary LF formation.

Uninfected *Cftr* ^*−/−*^ lung LF B cells also had significantly increased levels of CXCR4. CXCR4 is dynamically regulated during B cell maturation and plays an important role germinal center organization [24, 25]. CXCR4 responds to its ligand CXCL12 and its expression helps distinguish centroblasts from centrocytes. BAFF has not been reported to alter CXCR4 expression [35]. Whether increased CXCR4 expression in CFTR deficiency reflects an altered B cell maturation state and/or if it alters the ability of B cells to respond to germinal center chemokine gradients is unclear. These findings, in addition to increased lung B cell MHC class II expression, increased lung B cell BAFF and Ki67 expression, and increased inflammatory cytokine production upon activation raise concern for dysregulated immune responses to chronic infection and for the loss of immune tolerance to self-antigens that could lead to autoimmunity. Elevated autoantibody levels have been linked to lung disease severity in patients with CF [36, 37]. In addition, several systemic autoimmune disorders are also associated with CF including CF arthropathy [38–40] and cutaneous vasculitis [41].

The mechanisms by which CFTR-deficiency alters immune function in B cells have yet to be delineated. B cell chloride conductance is altered in CFTR deficiency [10, 11]. It has been postulated that altered lymphocyte chloride conductance could alter membrane potential and in turn calcium flux. T lymphocyte intracellular calcium flux has been shown to be increased in CFTR deficient T cells upon stimulation. Increased nuclear factor of activated T-cells (NFAT) nuclear translocation, which is modulated by calcium-associated signaling pathways, was observed in *Cftr* ^*−/−*^ T cells upon activation resulting in increased inflammatory cytokine production [8].

Interactions between CFTR and other molecules important in immune activation may also be important. Activated CFTR deficient macrophages have reduced ezrin protein levels and altered localization [28]. Ezrin is a member of the ezrin-radixin-moesin family that bridges plasma membrane proteins to the actin cytoskeleton and regulates cellular processes that require membrane remodeling and modulate signaling events. Decreased ezrin levels were linked to reduced PI3K/AKT signaling upon TLR4 activation in CFTR deficient macrophages and promoted a pro-inflammatory phenotype [28]. Ezrin has also be shown to maintain the topology of signaling molecules in the immunologic synapse and to down regulate Erk1/2 and NFAT signaling [42] in T lymphocytes. Ezrin plays a critical role in regulating B cell receptor signaling and lipid raft aggregation. Loss of ezrin increases B cell activation and increased MHC class II expression [42]. Reduced ezrin levels, as have been observed in CFTR deficient macrophages, may alter the function of the immunologic synapse and downstream signaling contributing to pro-inflammatory responses in CFTR deficient B cells as well. The role of these mechanisms in CFTR deficient B cells and their importance in the pathogenesis of CF remains to be defined.

In light of the chronic airway exposure to bacterial pathogens that occurs in cystic fibrosis, the expression of pathogen sensing molecules is likely an important contributor to immune activation. CFTR-dependent aberrant TLR4 trafficking has been observed in CFTR deficient macrophages, which promoted a hyperinflammatory response [4, 28]. B cell TLR4 expression was examined in CF subjects and controls. In contrast to mice, humans B cells do not constitutively express TLR4. However, human B cells have been shown to upregulate TLR4 in response to inflammatory stimuli [29]. As expected, based on literature, there was scant expression of TLR4 in lung B cells from control subjects. There was a significantly higher number of TLR4^+^ B cells in CF subjects (Fig. 6). Increased B cell TLR4 expression may amplify cytokine production as has been shown in macrophages. TLR4 expression can also contribute to B cell maturation. Interestingly, BAFF and TLR4, both of which had significantly increased expression in lung B cells in CF subjects, have been previously shown to have a synergistic effect on B cell maturation [27]. Additional studies will be needed to dissect the complex mechanisms that promote B cell dysregulation and lymphoid follicle formation in cystic fibrosis.

The limitations of this study include the fact that with few exceptions human tissue samples studied were from patients with severe lung disease. Lung biopsies are infrequently performed in patients with CF, so correlation of immunohistochemical findings with FEV1 and other clinical parameters over a spectrum of disease was not possible. Given that *Cftr* ^*−/−*^ mice do not develop the airway pathology observed in patients with cystic fibrosis, the effects of CFTR deficiency on B cell function and activation observed in murine analyses may be more pronounced or altered in human disease. We did not examine the ability of *Cftr* ^*−/−*^ B cells to promote humoral immunity and host defense as it was beyond the scope of this study. We also did not examine the diverse B cell subsets found in LFs and germinal centers. It is possible that CFTR deficiency may play a more prominent role in specific B cell maturation subsets. In addition, since archival lung tissue from patients with CF was used, correlation of immunohistochemical findings with B cell functional assays using peripheral blood B cells from patients with CF and controls could not be performed.

The data presented here point to an important role for B cells and tertiary LF development in CFTR-mediated immune activation and the pathobiology of lung disease in CF. Although we did not examine other organs typically affected by CF such as the gastrointestinal system, it is possible that altered B cell responses in CF could affect other sites of disease. Immune dysregulation may be an important contributor to the pathobiology of CF where chronic airway infection may augment the effects of CFTR-mediated immune dysfunction. There may be a clinical role for immunomodulation in altering CF outcomes. Finding biomarkers that identify immune dysregulation and identifying precise pharmacologic targets that can alter disease progression in CF merit further investigation.

## Conclusion

The CF transmembrane conductance regulator (CFTR) plays a direct role in promoting tertiary lung LF development, B cell activation and B cell inflammatory cytokine production in the absence of infection.

## Data Availability

The data sets generated and analyzed during this study are available from the contributing author upon request.

## Abbreviations

BAFF: B cell-activating factor of tumor necrosis factor family
CF: Cystic fibrosis
CFTR: Cystic fibrosis transmembrane conductance regulator
CXCR4: C-X-C chemokine receptor type 4
FEV1: Forced expiratory volume in 1 s
LFs: Lymphoid follicles
MHC class II: Major histocompatibility complex class II
NFAT: Nuclear factor of activated T-cells
